# Global Measles Surveillance: Trends, Challenges, and Implications for Public Health Interventions

**DOI:** 10.3390/idr16020028

**Published:** 2024-04-16

**Authors:** Francesco Branda, Marta Giovanetti, Chiara Romano, Domenico Benvenuto, Alessandra Ciccozzi, Daria Sanna, Massimo Ciccozzi, Fabio Scarpa

**Affiliations:** 1 Unit of Medical Statistics and Molecular Epidemiology, Università Campus Bio-Medico di Roma, 00128 Rome, Italy; chiararomanoduemila@gmail.com (C.R.); m.ciccozzi@unicampus.it (M.C.); 2 Department of Sciences and Technologies for Sustainable Development and One Health, Università Campus Bio-Medico di Roma, 00128 Rome, Italy; 3 Instituto René Rachou, Fundação Oswaldo Cruz, Belo Horizonte, Belo Horizonte 30.190-009, Brazil; 4 Climate Amplified Diseases and Epidemics (CLIMADE), Brazil; 5 Medical Statistic and Molecular Epidemiology Unit, Universita Cattolica di Roma, 00135 Rome, Italy; 6 Department of Biomedical Sciences, University of Sassari, 07100 Sassari, Italy; ale_ciccozzi97@icloud.com (A.C.); darsanna@uniss.it (D.S.); fscarpa@uniss.it (F.S.)

**Keywords:** measles, infectious diseases, public health, outbreak investigation, surveillance data, risk assessment, vaccination strategies, data analysis

## Abstract

Measles, a highly contagious disease primarily affecting children, carries serious health risks, including complications and mortality. Vaccination remains the most effective preventive measure against measles transmission. The COVID-19 pandemic has exacerbated challenges in surveillance and immunization efforts, leaving millions of people exposed to preventable diseases such as measles. Globally accelerated immunization campaigns are critical for achieving regional elimination goals and mitigating the risk of outbreaks. Our team has developed an open-access database for global measles monitoring, facilitating standardized data collection and analysis. The analysis of measles cases from 2011 to 2023 reveals fluctuating trends, with notable increases in Africa in 2019 and 2023, indicating potential gaps in control strategies. Using an automated signal detection tool developed by the European Centre for Disease Prevention and Control (ECDC) team, we identified significant variations between World Health Organization (WHO) regions, underscoring the importance of continuous monitoring to detect epidemiological changes early. These results underscore the need for robust surveillance systems and accelerated vaccination efforts to safeguard public health.

## 1. Introduction

Measles ranks among the most contagious viral diseases, and is caused by the measles virus (MeV), which is part of the Morbillivirus genus within the Paramyxoviridae family. Notably, its basic epidemiological reproductive rate (R0) far exceeds that of diseases like influenza. This indicates that a single individual with measles can infect, on average, 12–18 people in a fully susceptible population during normal social interactions [1,2]. Typically, children without specific immunity—usually provided by vaccination—are the most affected. Measles transmission primarily occurs through direct contact with infectious droplets or via airborne spread when an infected individual breathes, coughs, or sneezes. Airborne transmission is particularly notable, with infectious particles capable of remaining viable in indoor environments for up to 2 h, thereby significantly enhancing transmission dynamics. Initial symptoms, appearing 10–12 days after infection, include high fever, a runny nose, bloodshot eyes, and tiny white spots inside the mouth [3,4,5]. A rash emerges several days later, beginning on the face and upper neck before spreading downward. Severe cases of measles are more common in poorly nourished young children, particularly those with weakened immune systems due to significant diseases or specific immunomodulatory therapies. Serious complications, such as blindness, encephalitis, severe diarrhea leading to dehydration, and severe respiratory infections including bacterial and viral pneumonias, can arise from measles infections, sometimes resulting in death. While no specific treatment for measles exists, supportive care can significantly improve outcomes [1,5]. Despite the availability of safe and effective vaccines, measles remains a leading cause of child mortality globally, especially in regions with poor nutrition and inadequate healthcare systems. The vaccine, which is both safe and can be administered alongside other vaccines, led to an 80% reduction in measles-related deaths between 2000 and 2017. By 2017, approximately 85% of children worldwide had received their first dose of the vaccine. However, the disease and death rates saw an increase from 2017 to 2019 due to declining immunization rates [6,7]. This condition can be explained by the occurrence of various factors that have contributed to the decline in vaccination rates. For instance, the COVID-19 pandemic has notably played a significant role in worsening this trend. During this period, the spread of anti-vaccine messages likely fueled doubts and distrust towards vaccines. This dissemination of false information has led individuals to question vaccine safety and effectiveness, resulting in delays in vaccine uptake. In response, the World Health Organization (WHO) has initiated efforts to eliminate measles by strengthening immunization programs. This study aims to describe measles outbreaks in various countries to gain a clearer understanding of the current situation based on available data [8].

## 2. Materials and Methods

Our analysis involves basic signal detection to examine the total frequency of measles outbreaks across World Health Organization (WHO) regions, with additional details provided in the Supplementary File. The process begins with downloading measles cases by country and month from the WHO website: https://www.who.int/teams/immunization-vaccines-and-biologicals/immunization-analysis-and-insights/surveillance/monitoring/provisional-monthly-measles-and-rubella-data (accessed on 25 February 2024). Subsequently, we standardize these data in alignment with the Surveillance Atlas of Infectious Diseases [9], a platform facilitated by the European Centre for Disease Prevention and Control (ECDC) that enables interactive data manipulation regarding principal infectious diseases, collected through The European Surveillance System (TESSy). To identify significant changes in disease incidence and potential outbreaks effectively, we employ the EpiSignalDetection [10], an R package developed by ECDC biostatisticians. This package is based on sophisticated signal detection algorithms designed for the early recognition, investigation, and initiation of control measures. It includes two primary detection algorithms: (i) The Farrington Flexible utilizes range values from surveillance time series. For each time point, it employs a Poisson Generalized Linear Model (GLM) with overdispersion to predict an upper limit on count numbers, following the method outlined by Farrington et al. [11] and Noufaily et al. [12]. An alarm is triggered if the observed counts exceed this predicted upper bound, a technique extensively adopted by public health institutes [13]. (ii) The Generalized Likelihood Ratio statistic for Negative Binomial distribution (GLRNB) serves as a regression chart for count data in surveillance time series monitoring. This method, based on the generalized likelihood ratio (GLR) as detailed by Höhle and Paul [14], is specifically designed for analyzing seasonal count data. These algorithms are integral components of the surveillance R package [15], ensuring a comprehensive and timely approach to outbreak detection and response.

## 3. Results

### 3.1. Description of the Situation

**Figure 1:**
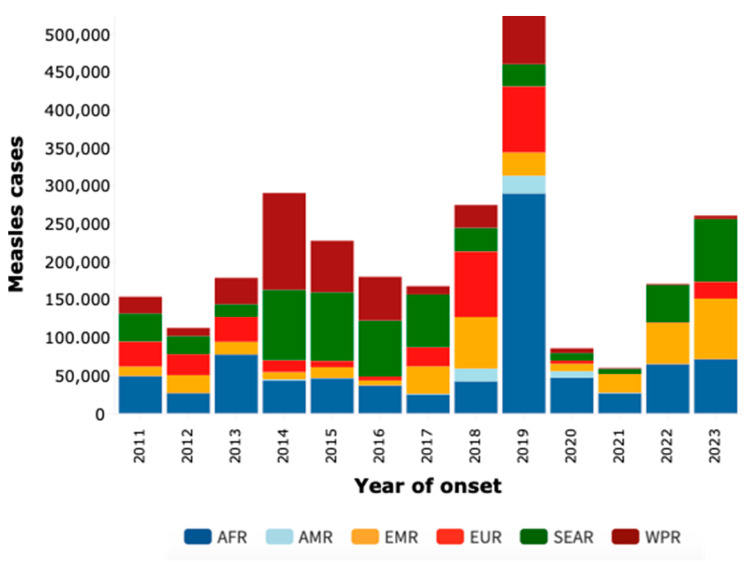
Measles case distribution by year and WHO region (2011–2023).

Our analysis of measles cases across World Health Organization (WHO) regions from 2011 to 2023, as illustrated in Figure 1, reveals fluctuating trends and significant variations. A notable surge in cases was observed in Africa in 2019, with nearly 290,000 cases, prompting questions regarding the sustainability of existing control strategies. In 2020, cases significantly dropped to 46,747, a decrease reflecting the impact of the COVID-19 pandemic on the dynamics of infectious disease transmission. The subsequent years, 2021 and 2022, exhibited a downward trend, potentially indicating the success of vaccination campaigns and prevention efforts. However, a concerning uptick in cases occurred in 2023, especially in Africa, where more than 71,000 cases were reported. This sharp increase highlights concerns over the effectiveness of long-term preventive measures. By December 2023, a total of 260,465 confirmed cases were reported to WHO through official monthly reports from member states. This total includes 71,384 cases from the African Region (AFR), 49 from the Americas Region (AMR), 79,688 from the Eastern Mediterranean Region (EMR), 22,137 from the EU/EEA Regions (EUR), 82,667 from the Southeast Asian Region (SEAR), and 4540 from the Western Pacific Region (WPR).

**Figure 2:**
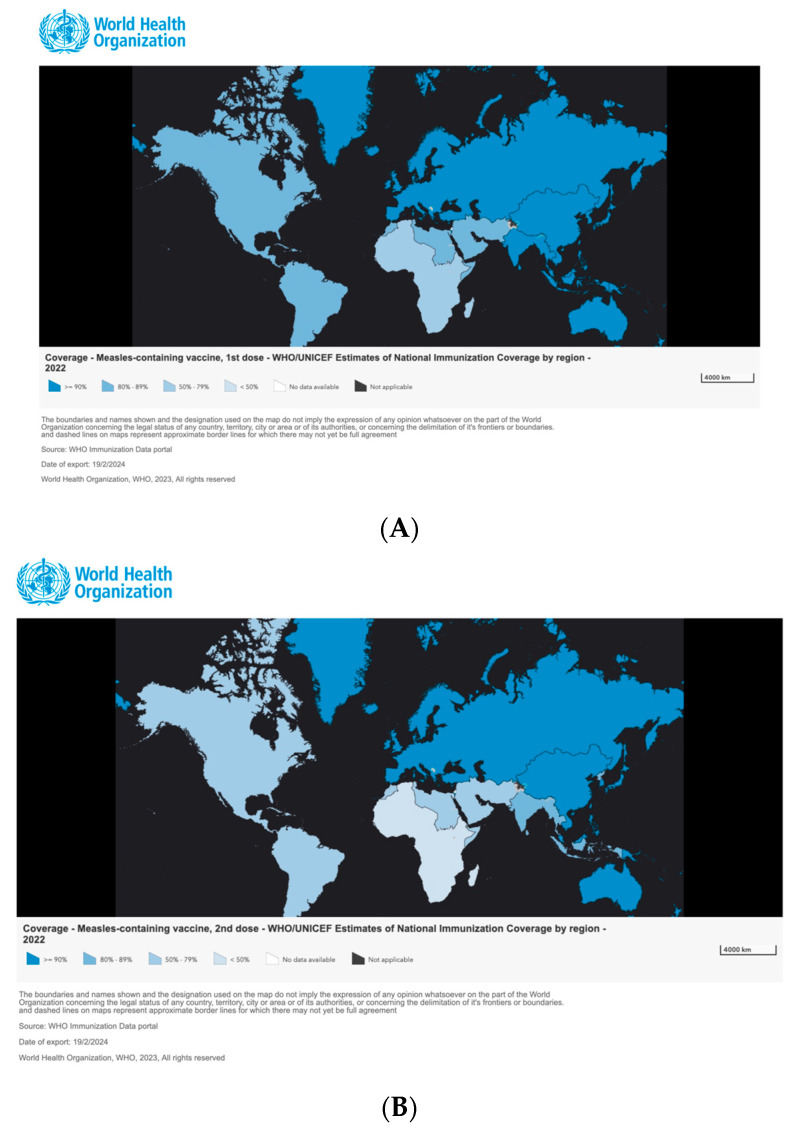
Measles vaccination coverage by WHO region (2011–2022): (**A**) estimates of national immunization coverage per 1st dose; (**B**) estimates of national immunization coverage per 2nd dose. Source: https://immunizationdata.who.int (accessed on 25 February 2024).

The analysis of measles immunization data across various World Health Organization (WHO) regions, as depicted in Figure 2, uncovers diverse trends and coverage levels. In the African Region (AFR), immunization coverage is particularly troubling, with the second dose of the measles vaccine (MCV2) falling to 45% in 2022, and the first dose (MCV1) at 69%. This represents the continuation of a downward trend, with MCV2 coverage having been even lower at 41% in 2021, indicating a regression in vaccination efforts and an increased risk of outbreaks. The Eastern Mediterranean Region (EMR) displays variability in its immunization coverage. In 2022, MCV2 coverage was at 75%, with MCV1 at 86%. Although these rates surpass those observed in AFR, there was a decrease from the 79% MCV2 coverage in 2021, suggesting some fluctuations in vaccine uptake. Conversely, the European Region (EUR) shows promising signs, with high levels of measles immunization. MCV2 coverage reached 91%, and MCV1 was at 93% in 2022, despite slight decreases from the previous year, indicating robust vaccination programs. The Americas Region (AMR) reveals a concerning decline in vaccine coverage over time. In 2022, MCV1 coverage dropped to 84%, and MCV2 was at 76%, marking a notable decrease from previous years and highlighting challenges in maintaining immunization rates. The Southeast Asian Region (SEAR) also experiences a general downward trend, especially for MCV2. In 2022, MCV1 coverage was 92%, but MCV2 fell to 85%, pointing to areas for improvement in vaccination efforts. In stark contrast, the Western Pacific Region (WPR) maintains consistently high and stable vaccination coverage. In 2022, both the MCV1 and MCV2 coverage rates stood firm at 91%, reflecting sustained success in measles vaccination programs.

The following is a brief update on the global measles situation based on information shared by member states with WHO. Note that this is an evolving situation and data are constantly updated. The data below are based on the latest information available to WHO.

### 3.2. African Region

Several countries in the region reported large measles outbreaks, demonstrating a diverse and dynamic situation. For example, Algeria has maintained relative stability with a low numbers of cases, in sharp contrast to Angola, which experienced a sharp increase to 12,009 cases in 2014, when MCV1 coverage was only 56% and MCV2 had not been introduced, followed by a dramatic decline to 103 cases in 2015, when MCV2 coverage reached 16%. However, in 2022, with MCV1 at 37% and MCV2 at 25%, cases increased again to 3267. The situation in Burkina Faso reveals a steady increase in measles cases, with a notable peak in 2022 at 1019 cases, despite maintaining a relatively high MCV1 coverage of around 88% but a low MCV2 coverage of 71% during this period. Burundi, after years of relative stability with an MCV1 coverage of around 90% but a gradual improvement in MCV2 from 51% in 2013 to 85% in 2022, experienced a sudden surge in cases starting in 2020, peaking at 1701 cases. Cameroon shows a more complex trend, with significant peaks in 2019 and again in 2023, despite having an MCV1 coverage of around 60–65% and introducing MCV2 with coverage reaching 44% in 2022. Central African Republic saw a significant reduction in cases in 2020, down to eight, likely influenced by global pandemic control measures, with MCV1 coverage at 41%.

### 3.3. Eastern Mediterranean Region

The measles situation across various countries demonstrates a range of trends and challenges, reflecting the impact of different factors such as vaccination coverage, public health responses, and socio-political contexts. In Afghanistan, despite an increase in coverage for the first dose from levels below 70% in 2011–2015 to around 68% in 2022, measles cases rose sharply to over 5000 in 2022, before declining slightly in 2023. Coverage for the second dose remained low at around 49% in 2022, highlighting the need to strengthen both doses. Bahrain’s case shows excellent vaccine coverage, with rates above 99% for both doses, consistent with the very low number of reported cases. In Djibouti, the increase in cases since 2022 appears to be associated with a decline in vaccine coverage, with the first dose dropping to 50% and the second dose to 48% in 2021–2022. Egypt has maintained relatively high vaccine coverages, above 90% for both doses, likely contributing to the decline in cases since the 2015 peak. Iran has seen extremely high coverages, above 98% for both doses, which may explain the low levels of cases despite a peak in 2015. Iraq experienced an increase in cases in 2023 despite increased vaccine coverage, with the first dose at 88% and the second at 97% in 2022, suggesting that additional control measures may be needed. Lebanon experienced a spike in cases in 2018, followed by a decline, during a period when vaccine coverage remained relatively stable at around 82% for the first dose and 63% for the second dose. In Libya, fluctuations in cases appear to correspond to significant changes in vaccine coverages, with a decline to 73% for both doses in 2018–2022. Pakistan has faced continued challenges, with case spikes in 2011, 2013, 2017, and 2023, despite gradual increases in vaccine coverage, reaching 82% for the first dose and 79% for the second dose in 2022. Saudi Arabia has maintained very high vaccine coverages, above 95% for both doses, consistent with low levels of reported cases. In Somalia, the severe spike in cases in 2017 could be related to gaps in vaccination coverage, for which no data are available. Sudan saw a decline in cases in 2020, followed by an increase in 2021 and 2023, despite a relatively stable vaccine coverage of around 81% for the first dose, but a lower coverage for the second dose, around 63% in 2022. Syria experienced fluctuations in cases, reflecting the difficulties of managing disease in a crisis context, with declining vaccination coverages, down to 41% for the first dose and 38% for the second in 2022. The United Arab Emirates maintained excellent vaccination coverages, above 90% for both doses, in line with the low levels of reported cases. Yemen has faced a critical situation, with significant increases in cases in 2011, 2018, 2021, and 2022, despite a gradual increase in vaccine coverages, reaching 73% for the first dose and 56% for the second in 2022.

### 3.4. European Region

The analysis of measles cases in Europe from 2011 to 2023 unveils diverse trends, underscoring the complexities of managing this infectious disease across the region. For example, Albania experienced a significant peak in 2018 with 1466 cases, but saw a significant decline in subsequent years, dropping to 482 cases in 2019 and only 4 in 2020. In the period between 2011 and 2022, it generally maintained MCV1 vaccine coverage above 90%, ranging from 94% in 2018 to 86% in 2022, while MCV2 vaccine coverage was generally higher, hovering around 99% in many years. In Armenia, a significant increase in measles cases was observed in 2013 and 2014, followed by a decrease in subsequent years, culminating in a significant decline in 2020 and no cases reported in 2021. This pattern suggests the success of enhanced immunization strategies and increased public awareness in combating the disease. For example, MCV1 vaccine coverage in Armenia has remained consistently high, fluctuating between 97% and 94% from 2011 to 2022, while MCV2 vaccine coverage has remained between 98% and 94% over the same period. In contrast, countries such as Austria and Bosnia and Herzegovina have seen a steady increase in measles cases over the years. Austria saw a spike in cases in 2015, while Bosnia and Herzegovina faced significant outbreaks in 2014 and 2015, followed by a slow decline. These cases indicate the need to look more closely at the immunization policies and transmission mechanisms of measles within these countries. For example, Austria maintained MCV1 vaccine coverage between 84% and 99% from 2011 to 2022, while MCV2 vaccine coverage was more variable, ranging between 73% and 94%. Furthermore, countries such as Italy and France have encountered significant outbreaks in recent years. In particular, Italy faced a significant measles outbreak in 2017, reporting 5399 cases. However, MCV1 vaccine coverage in Italy remained generally high, with values ranging from 90% to 94% from 2011 to 2022. MCV2 vaccine coverage, although more variable, has remained high overall, with values ranging from 82% to 89%. France experienced a notable outbreak in 2011, with 14,966 cases reported. Despite this, vaccine coverage in France remained robust over time, with MCV1 vaccine coverage ranging between 89% and 94%, while MCV2 vaccine coverage remained between 67% and 90% during the same period.

### 3.5. Region of the Americas

In the North American Region, measles cases showed significant fluctuations from 2011 to 2023. Countries such as Antigua and Barbuda, Bahamas, Costa Rica, Cuba, and Guatemala generally reported few or no cases during this period. However, some countries experienced severe outbreaks, particularly the United States and Canada. The United States reported peaks of 1282 cases in 2019 and 668 cases in 2014, while Canada reported 418 cases in 2014 and 113 in 2019. MCV1 coverage was generally high, with several countries maintaining rates above 90% for much of the period. However, there were some exceptions such as Guatemala, which had an MCV1 coverage below 80% in several years. For MCV2, coverages have been more variable and often lower than those of the first dose. Several countries, such as Mexico, Guatemala, and Saint Lucia, have struggled to maintain MCV2 coverage above 95%, the recommended threshold for measles control.

Several South American countries experienced major measles outbreaks, with Brazil having the highest peak of 20,001 cases in 2019, followed by Venezuela with 712 cases in 2017 and 548 in 2018. Colombia also had a surge of 244 cases in 2019. Coverages of the first dose of measles vaccine were generally good in the region, with most countries maintaining rates above 80% and often above 90%. However, there have been some exceptions such as Venezuela, which has had MCV1 coverages below 70% in several years. On the other hand, coverages of the second dose have been more variable and often falling short of the recommended threshold of 95%. Countries such as Argentina, Brazil, Chile, Colombia, Ecuador, and Venezuela had MCV2 coverages below 80% in several years, potentially contributing to the observed outbreaks.

### 3.6. South East Asia Region

The measles situation across various Asian countries demonstrates a broad spectrum of trends, indicating the diverse challenges in managing this infectious disease. In Bangladesh, a notable peak of 4001 cases in 2017 was followed by a decline, but a resurgence to 203 cases in 2021 highlights the need for continued vigilance. High and consistent coverage rates for both the first and second doses of the measles vaccine (MCV1 and MCV2) have been observed over the years, with MCV1 coverage consistently above 90% and MCV2 above 90% in recent years. This robust vaccine coverage has helped to keep the number of measles cases relatively low, with occasional small outbreaks. However, the slight decline in MCV2 coverage in 2021 raises concern and underscores the need for continued efforts to keep vaccination rates high. Bhutan presents a comparatively stable scenario, with 70 cases reported in 2017 and minimal numbers thereafter, underscoring the need for continued attention even in countries with historically low numbers of cases. In addition, Bhutan has demonstrated commendable performance in measles vaccination, with consistently high coverage rates for both MCV1 and MCV2, often exceeding 95%. This sustained effort has helped to keep measles cases in the country minimal, highlighting the effectiveness of vaccination programs in preventing outbreaks. The Democratic People’s Republic of Korea reported a minimal number of cases in 2013, with no significant reports in subsequent years, although the possibility of under-reporting exists. Nevertheless, the DPRK has maintained exceptionally high coverage rates for MCV1 over the years, consistently above 98%. However, in 2021 and 2022 there was a worrying decline in coverage for MCV1, which alerts to potential gaps in vaccination efforts. In addition, although MCV2 coverage has shown improvement, reaching 99% in recent years, there is still room for improvement to ensure complete protection against measles. India experienced a significant decrease in measles cases from 2014 to 2019, but witnessed an increase in 2020 and 2023, with the latter year marking the highest count with 69,486 cases, illustrating the challenges of measles control in densely populated nations. It also faced significant challenges in measles control due to its large population and diverse geographic landscape. Although MCV1 coverage has improved over the years, reaching 95% in 2022, there have been fluctuations, with occasional declines below 90%. MCV2 coverage, on the other hand, has been consistently lower, hovering around 80–90%, indicating the need for targeted interventions to increase second-dose immunization rates and improve overall measles protection. Indonesia was able to effectively control the situation in 2017 and 2018; however, an increase in cases in 2019 and 2020, followed by a decrease in 2021 and another increase in 2023, indicates ongoing challenges in achieving stabilization. Fluctuations in measles vaccination coverage have been observed in Indonesia, with MCV1 coverage showing a slight decline in recent years, dropping to 84% in 2022. Similarly, MCV2 coverage has shown some variability, with rates ranging from 50% to 71% in different years. These fluctuations underscore the importance of maintaining consistent vaccination efforts to prevent the resurgence of measles cases.

Sri Lanka had effectively managed measles until 2013, but experienced an uptick in 2014, subsequently followed by a decrease. Sri Lanka has demonstrated exemplary performance in measles vaccination, with consistently high coverage rates for both MCV1 and MCV2, often exceeding 98%. This sustained effort has led to minimal occurrences of measles cases in the country, highlighting the effectiveness of vaccination programs in preventing outbreaks. Thailand achieved a significant reduction in cases in 2019, yet observed new cases in 2020 and 2023, highlighting the need for continuous monitoring. It has maintained commendable coverage rates for both MCV1 and MCV2, with MCV1 coverage consistently above 95% and MCV2 coverage above 87% in recent years. These efforts have contributed to maintaining low measles case counts, although there is a need for continued vigilance to sustain high vaccination rates and prevent outbreaks. Timor-Leste’s situation has shown significant variations, with critical moments in 2011, 2014, and 2019, highlighting the importance of long-term strategies for measles control. Despite these challenges, Timor-Leste has made remarkable progress in measles vaccination. First-dose coverage of the measles vaccine (MCV1) has shown improvement in recent years, reaching 79% in 2022. Similarly, coverage of the second dose (MCV2) has increased to 78% in 2022. These efforts have helped reduce the impact of measles in the country; however, continued work is needed to increase vaccination rates and ensure comprehensive protection against this disease.

### 3.7. Western Pacific Region

Australia experienced a surge in measles cases in 2014 with 340 cases, followed by a dramatic reduction to only 26 cases in 2020 and no cases in 2021. However, a slight increase was observed in subsequent years, with 7 cases reported in 2022 and 26 in 2023, underscoring the need for continued vigilance. In the face of this, Australia has maintained consistently high vaccination coverage, with rates for the first dose of measles vaccine reaching 96% in 2022 and for the second dose declining slightly to 91% in the same year, highlighting a sustained commitment over time to prevention of the disease. Brunei Darussalam offers a contrasting scenario, with very few cases reported since 2011 and no cases in most subsequent years, demonstrating the success of its control and vaccination strategies in almost completely eliminating measles. The country has achieved and maintained impressive vaccination coverage, with coverage for MCV1 at 97% and for MCV2 at 99% in 2022, consolidating its success in the fight against measles. In Cambodia, the situation has been variable, with a notable increase in 2011 (722 cases), years when no cases were reported, and then another spike in 2019 (675 cases), underscoring the importance of sustained efforts to manage the disease. Despite the challenges, vaccine coverage for MCV1 remained relatively stable at about 83% in 2022, while coverage for MCV2 showed significant improvements, reaching 69% in the same year, highlighting progress toward greater protection from measles. China experienced a significant number of cases in 2011 (9943), but has seen a steady decline, thanks to exceptionally high vaccine coverage, with both doses of the vaccine (MCV1 and MCV2), maintaining 99% coverage over the years, reflecting a strong commitment to measles control. Mongolia faced a dramatic increase in cases in 2015 (20,636), with declining numbers in subsequent years, reflecting the impact of specific events or vaccination strategies. The country has maintained high vaccination coverage, with MCV1 at 94% and MCV2 at 93% in 2022, contributing to the decrease in measles cases. Malaysia has experienced an upward trend in measles cases, with peaks in 2011 (1608 cases) and 2018 (1958 cases). Despite a decline in 2020 (477 cases), vaccination coverage remained high, with MCV1 at 96% and MCV2 at 96% in 2022, underscoring the importance of improved patient safety.

### 3.8. Signal Detection Analysis

**Figure 3:**
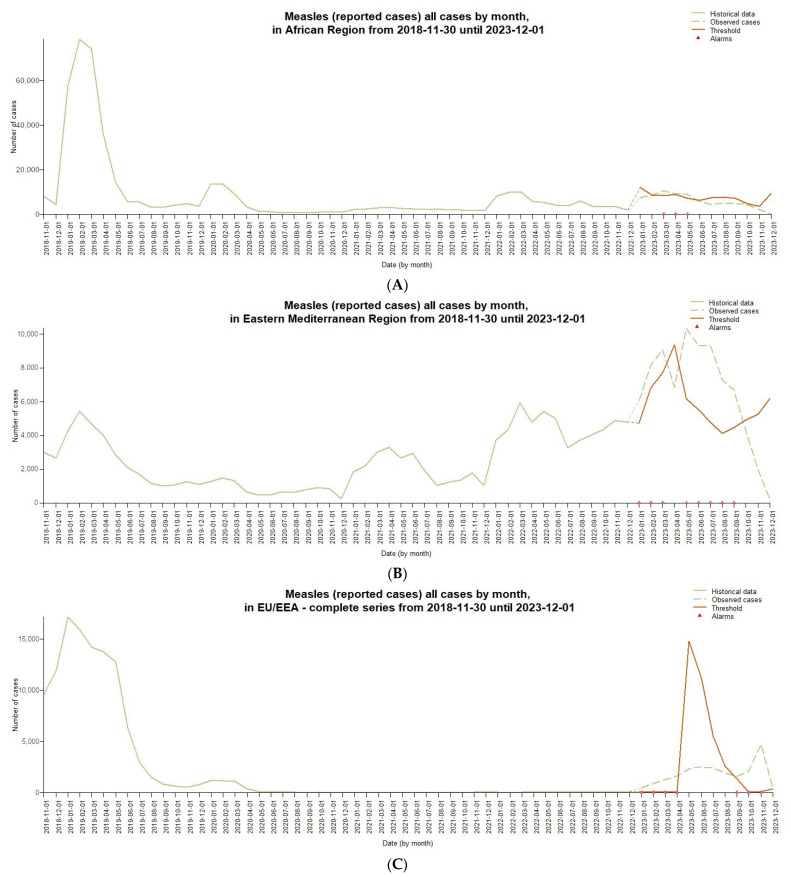

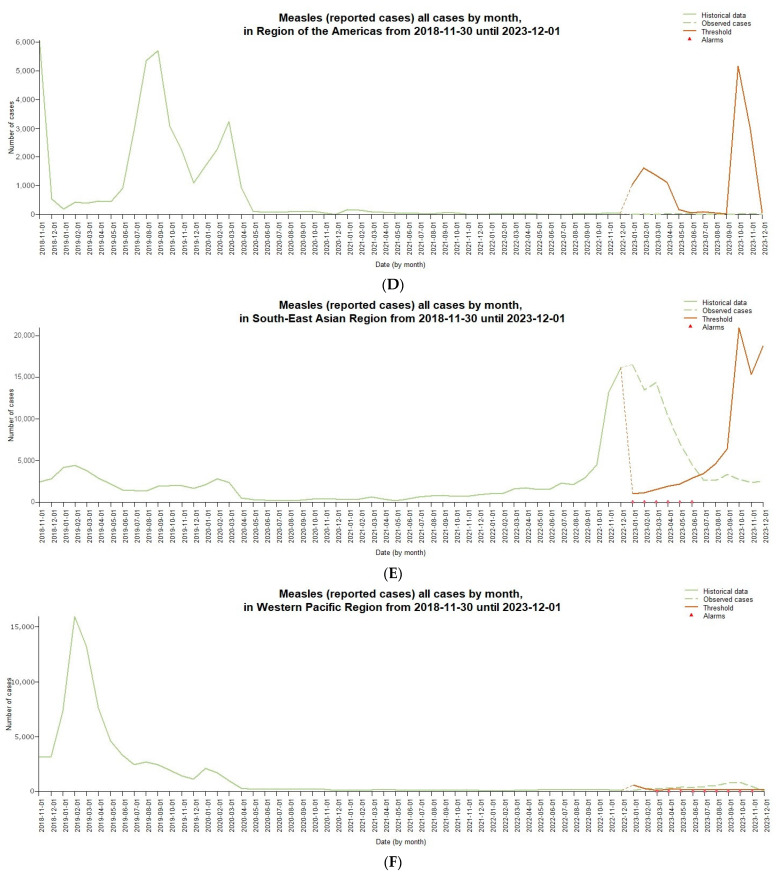
Example of using the Farrington Flexible algorithm for monitoring measles disease surveillance data: (**A**) AFR; (**B**) EMR; (**C**) EUR; (**D**) AMR; (**E**) SEAR; (**F**) WPR.

The signal detection analysis shown in Figure 3 aggregates measles cases reported by month in different WHO regions from 2018 to the end of 2023. The graphs include historical data with a light green dashed line used for prediction by the Farrington Flexible algorithm, cases observed during the signal detection period with a solid green line, a threshold value with a dark orange dashed line, and detected signals with red triangles, indicating potential anomalies or changes in trends.

Analyzing regional trends, measles cases in the African Region (Figure 3A) showed a steady increase until March 2023, when positive signs of potential abnormalities were detected with monthly spikes of more than 10,000 cases. However, from April to July 2023, cases gradually declined, falling below the expected threshold. In the last quarter of the year, a significant decline was observed with only 247 cases in December, suggesting a possible change in the epidemiological scenario. Consistent positive signs also occurred in Eastern Mediterranean countries and territories (Figure 3B) during 2023, with peaks of more than 10,000 cases monthly from March to July. After a decline in August–October, cases increased again at the end of the year, suggesting a prolonged and persistent epidemic underway. In Europe (Figure 3C), after relative stability in 2022, there was a significant increase in cases beginning in January 2023, with positive signs persisting for several months. The highest peaks were recorded in April (1634 cases) and November (4732 cases), indicating possible ongoing outbreaks. Only in December 2023 did cases return below the expected threshold. In the Americas Region (Figure 3D), the number of monthly cases remained relatively low and contained throughout 2023, with no signs of potential anomalies detected by the algorithm. The highest values were just 12 cases in November. The Southeast Asian Region (Figure 3E) showed positive signs of abnormalities as early as January 2023, with a very high number of monthly cases, exceeding 10,000 from March to June. Although decreasing in the following months, the levels still remained significant until December, indicating a critical and widespread epidemiological situation. In the Western Pacific Region (Figure 3F), there is evidence of a significant upward trend in cases since March 2023, with consecutive positive signs through November. The highest peak was 838 cases in October, suggesting a possible ongoing epidemiological emergency.

### 3.9. Limitations

The limitations of this study include the following:

- Data Limitations: This study’s reliance on secondary data sources such as WHO reports or surveillance databases may introduce limitations related to data quality, consistency, and timeliness. The accuracy and completeness of these secondary sources can vary, potentially affecting the robustness of the study’s conclusions.
- Lack of data granularity: In the context of measles surveillance, data granularity could include factors such as age-specific incidence rates, vaccination coverage rates in specific populations, or detailed information on outbreak locations. Greater data specificity could address various aspects, such as age-specific incidence rates, vaccination coverage rates in particular populations, or detailed information on outbreak locations. A lack of detail prevents more in-depth analyses and identification of subgroups particularly at risk of measles outbreaks. For example, the absence of age-specific data makes it difficult to assess the effectiveness of vaccination campaigns on different age groups or to tailor preventive measures. In addition, the lack of detailed data limits the ability to identify specific geographic areas or communities in need of targeted interventions.
- Under-reporting or misclassification of measles cases: This may occur for various reasons, such as limited access to health facilities, misdiagnosis of measles symptoms, or difficulties in accurately documenting and reporting cases. Under-reporting can lead to an underestimation of the true incidence of measles, which can affect the accuracy of surveillance data and subsequent analysis. Misclassification of cases, when measles cases are identified or misclassified as other diseases, can also introduce errors into the data and affect the reliability of the study results.

## 4. Discussion

### 4.1. Urgency of Intensified Immunization Efforts

Measles is a highly contagious disease that affects individuals of all ages but is most prevalent among children. It spreads through respiratory droplets when an infected person breathes, coughs, or sneezes, leading to severe illness, complications, and even death. Vaccination is the most effective means to prevent measles infection and its spread. The vaccine is safe and equips the body to combat the virus. However, for full and adequate immunity, two doses of the vaccine are recommended, yet a significant number of individuals have only received the first dose. This partial vaccine coverage may contribute to significant mutations in the virus due to partial selective pressure. An interesting study recently published describes a significant mutation in some human measles isolates [16], highlighting how continuous viral circulation without comprehensive vaccination control can lead to mutations that may bypass standard antigen tests, underscoring the importance of a thorough vaccination strategy. The COVID-19 pandemic has disrupted global surveillance and vaccination efforts, leading to a decline in vaccination rates worldwide and leaving millions of children vulnerable to diseases like measles. Measles, which is highly globalized, can easily spread from high-incidence areas to regions with low vaccine coverage, potentially causing epidemics. Accelerated immunization efforts are crucial to reduce child mortality and meet regional measles elimination targets. It is imperative to restore lost progress due to the pandemic, reinforce immunization programs, and ensure widespread access to the measles vaccine, along with robust surveillance to address immunity gaps.

### 4.2. Analysis of Measles Cases and Vaccination Coverage

To aid global efforts in monitoring and tracking measles outbreaks, our team has developed an open-access database that compiles data on global cases and vaccination rates. This database not only makes data readily accessible to researchers and health workers but also supports the analysis and monitoring of global measles trends. It adheres to the ECDC model to utilize its automatic signal detection tool, facilitating early identification of abnormalities or concerns in measles spread and enabling prompt preventative and control measures in response to incidence rate changes. Our analysis of measles cases in WHO regions from 2011 to 2023 highlights fluctuating trends. For example, Africa experienced a surge in cases in 2019, reaching nearly 290,000, questioning the sustainability of control strategies. However, there was a significant decrease in 2020 to 46,747 cases due to the impact of the COVID-19 pandemic on disease transmission dynamics. Although successful vaccination campaigns and prevention efforts led to a downward trend in 2021 and 2022, the sharp increase in 2023, especially in Africa with over 71,000 cases, raises concerns about the effectiveness of long-term preventive measures. Vaccination coverage and trends across various WHO regions present differing scenarios. The downward trend in Africa is concerning, indicating a regression in vaccination programs and increasing vulnerability to outbreaks. While coverage rates in the Eastern Mediterranean Region (EMR) are higher, the decrease in the coverage of the second dose of the measles vaccine (MCV2) over the years highlights the need for ongoing efforts to ensure vaccine uptake and program effectiveness. In contrast, Europe (EUR) shows consistently high measles immunization levels, suggesting strong vaccination programs and public health initiatives. The Americas (AMR) and Southeast Asia (SEAR) regions face challenges with declining vaccination coverage, underscoring the importance of overcoming barriers to vaccine access and uptake. The Western Pacific Region (WPR), however, maintains stable and high vaccine coverage, indicating the successful implementation of immunization programs. Analyses using an automatic signal detection tool provided detailed insights into temporal changes across different epidemiological regions. In Africa, there was a steady upward trend until January 2023, with a subsequent negative signal suggesting a potential change. The Eastern Mediterranean Region (EMR) experienced a gradual increase, stabilizing by 2021, but a positive signal in January 2023 hinted at a possible anomaly. Similarly, European data remained relatively stable until January 2023, when positive signals indicated an unexpected increase. The AMR, SEAR, and WPR regions showed considerable variation, with key signals becoming evident in January 2023, indicating potential heterogeneity in epidemiological trends.

## 5. Conclusions

This study emphasizes the importance of constant monitoring and careful analysis of epidemiological data to identify significant changes in infectious disease spread patterns early. Early detection of changes in epidemiological trends is crucial for timely preventive and control measures to limit disease spread and mitigate public health impacts. Moreover, this work highlights the critical need to maintain and strengthen measles vaccination programs globally, address coverage disparities, and ensure equitable vaccine access to prevent outbreaks and protect public health. Continued investments in vaccination infrastructure, community engagement, and surveillance systems are essential to sustain progress and achieve measles elimination goals.

## Data Availability

The data that support the findings of this study are openly available at https://github.com/fbranda/measles (accessed on 25 February 2024).

## Supplementary Materials

The following supporting information can be downloaded at: https://www.mdpi.com/article/10.3390/idr16020028/s1. References [11,12,17,18,19] are cited in the Supplementary Materials.
