# Supplementary material for: Global Measles Surveillance: Trends, Challenges, and Implications for Public Health Interventions
Source: Infect Dis Rep. 2024 Apr 16;16(2):367–79. doi: 10.3390/idr16020028 (PMC11050389; doi:10.3390/idr16020028)
Supplement: Supplementary file 1 [file idr-16-00028-s001.zip › idr-2913339-supplementary.pdf]

Global Measles Surveillance and Immunization Strategies: Trends, Challenges, and Implications for Public Health

Francesco Branda[1,*], Marta Giovanetti[2,3,4,*], Chiara Romano[1], Domenico Benvenuto[5], Alessandra Ciccozzi[6], Daria Sanna[6], Massimo Ciccozzi[1], Fabio Scarpa[6]

1 Unit of Medical Statistics and Molecular Epidemiology, University Campus Bio-Medico of Rome, Rome, Italy
2 Instituto René Rachou, Fundação Oswaldo Cruz, Minas Gerais, Brazil
3 Climate Amplified Diseases and Epidemics (CLIMADE), Brazil, Americas
4 Sciences and Technologies for Sustainable Development and One Health, Universita Campus Bio-Medico di Roma, Italy
5 Universita Cattolica di Roma, Rome, Italy
6 Department of Biomedical Sciences, University of Sassari, Sassari, Italy

* Correspondence: f.branda@unicampus.it, giovanetti.marta@gmail.com

1. Dataset to be used in the Epidemiological Signal Detection Tool

The user interface of the signal detection tool is made available once the **EpiSignalDection** package is installed, and by running the following commands in the R console:

```
install.packages("EpiSignalDetection")
library(EpiSignalDetection)
EpiSignalDetection::runEpiSDApp()
```

Figure S1: R commands to run signal detection tool for monitoring infectious diseases in TESSy

A description of the main fields in our database is shown below:

Variables	Definition	Format
**HealthTopic**	Disease name	String
**Population**	Population characteristics (e.g., All cases, Confirmed cases, etc.)	String
**Indicator**	Indicators available (e.g., Hospitalized cases, Reported cases, Number of deaths, etc.)	String
**Unit**	Unit of the NumValue variable. 'N' stands for number of cases	String
**Time**	Dates in any formats available in the tool, i.e., yearly data %Y (e.g., 2023), monthly data %m (e.g., 2023-01) or weekly data in ISO week format (e.g., 2023-W10)	String
**RegionCode**	Geographical level in coded format	String
**RegionName**	Geographical level including full country names (e.g.Austria, Belgium, Bulgaria, etc.)	String
**NumValue**	Number of cases	Numeric

Table S1: Dataset specification (naming, format, description).

HealthTopic	Population	Indicator	Unit	Time	RegionCode	RegionName	NumValue
Measles	All cases	Reported cases	N	2023-10	AFR	African Region	4387
Measles	All cases	Reported cases	N	2023-11	AFR	African Region	2071
…	…	…	…	…	…	…	...
Measles	All cases	Reported cases	N	2023-10	EUR	EU/EEA - complete series	2097
Measles	All cases	Reported cases	N	2023-11	EUR	EU/EEA - complete series	4732
…	…	…	…	…	…	…	...
Measles	All cases	Reported cases	N	2023-10	AMR	Region of the Americas	7
Measles	All cases	Reported cases	N	2023-11	AMR	Region of the Americas	12
…	…	…	…	…	…	…	...

Table S2: Example of our data imported in the ECDC Atlas.

2. Setting parameters for the analysis

The specific values of the parameters used for each algorithm function are set according to the parameter table included in the package. Specifically, the parameters for the Farrington Flexible algorithm are as follows [3]:

- **w**: Window's half-size, i.e., number of weeks to include before and after the current week in each year.
- **trend**: Boolean indicating whether a trend should be included and maintained if the conditions in the paper by Farrington et. al. [1] are met (see results). If false, no trend is included.

- **weightsThreshold**: Defines the threshold for reweighting past outbreaks using the Anscombe residuals (1 in the original method [1], 2.58 advised in the improved method [2]).
- **glmWarnings**: Boolean specifying whether to print warnings from the call to *glm*, i.e., a function used to fit generalized linear models, specified by giving a symbolic description of the linear predictor and a description of the error distribution.
- **pThresholdTrend**: Threshold for deciding whether to keep trend in the model (0.05 in the original method [1], 1 advised in the improved method [2]).
- **limit54**: Vector containing two numbers: *cases* and *period*. To avoid alarms in cases where the time series only has about almost no cases in the specific week the algorithm uses the following heuristic criterion (see Section 3.8 of the Farrington paper [1]) to protect against low counts: no alarm is sounded if fewer than *cases*=5 reports were received in the past *period*=4 weeks.

The GLRNB algorithm, on the other hand, requires the following parameters [4]:

- **mu0**: A vector of in-control values of the mean of the Poisson / negative binomial distribution with the same length as *range*. If *NULL* the observed values in 1:(min(range)-1) are used to estimate the beta vector through a generalized linear model. To fine-tune the model one can instead specify mu0 as a list with two components: (i) *S*: integer number of harmonics to include (typically 1 or 2); (ii) *trend*: a boolean indicating whether to include a term *t* in the GLM model.
- **theta**: if *NULL* then the GLR scheme is used. If not *NULL* the prespecified value for κ
- or λ is used in a recursive LR scheme, which is faster.
- **alpha**: The (known) dispersion parameter of the negative binomial distribution, i.e. the parametrization of the negative binomial is such that the variance is $mean + alpha * mean2$ Note: This parametrization is the inverse of the shape parametrization used in R -- for example in dnbinom and glr.nb. Hence, if alpha=0 then the negative binomial distribution boils down to the Poisson distribution and a call of algo.glrnb is equivalent to a call to algo.glrpois. If alpha=NULL the parameter is calculated as part of the in-control estimation. However, the parameter is estimated only once from the first fit. Subsequent fittings are only for the parameters of the linear predictor with alpha fixed.

- **cARL**: threshold in the GLR test, i.e. $c\gamma$
- **Mtilde**: number of observations needed before we have a full rank the typical setup for the "*intercept*" and "*epi*" charts is Mtilde=1
- **M**: number of time instances back in time in the window-limited approach, i.e. the last value considered is max $1, n-M$. To always look back until the first observation use M=-1.
- **Change**: a string specifying the type of the alternative. Currently the two choices are *intercept* and *epi*.

The implementation of both algorithms is illustrated in Salmon et al. [5].

References

1 Farrington CP, Andrews NJ, Beale AD, Catchpole MA. A statistical algorithm for the early detection of outbreaks of infectious disease. Journal of the Royal Statistical Society: Series A (Statistics in Society). 1996 May;159(3):547-63.

2 NOUFAILY, Angela, et al. An improved algorithm for outbreak detection in multiple surveillance systems. Statistics in medicine, 2013, 32.7: 1206-1222.

3 Meyer S. Surveillance for univariate count time series using an improved Farrington Method [Accessed: 3 April 2023]. Available from: https://surveillance.r-forge.r-project.org/pkgdown/reference/farringtonFlexible.html

4 Meyer S. Count data regression charts - algo.glrnb [Accessed: 3 April 2023]. Available from: https://surveillance.r-forge.r-project.org/pkgdown/reference/algo.glrnb.html

5 Maëlle S, Dirk S, Michael H. Monitoring count time series in R: Aberration detection in public health surveillance. arXiv preprint arXiv:1411.1292. 2014 Nov 5.
